# Profiling the Bladder Microbiota in Patients With Bladder Cancer

**DOI:** 10.3389/fmicb.2021.718776

**Published:** 2022-02-07

**Authors:** Mónica Parra-Grande, Martín Oré-Arce, Llúcia Martínez-Priego, Giuseppe D’Auria, Ramón Rosselló-Mora, Marta Lillo, Andrea Sempere, Blanca Lumbreras, Victoria Sánchez-Hellín

**Affiliations:** ^1^Department of Microbiology, Hospital General Universitario de Elche, Elche, Spain; ^2^Departament of Oncology, Hospital Marina Baixa de La Vila Joiosa, Villajoyosa, Spain; ^3^Sequencing and Bioinformatics Service, Foundation for the Promotion of Health and Biomedical Research of Valencia Region, FISABIO, Valencia, Spain; ^4^Marine Microbiology Group, Institut Mediterrani d’Estudis Avançats (CSIC-UIB), Esporles, Spain; ^5^Biobank, Hospital General Universitario de Elche, Elche, Spain; ^6^Departament of Public Health, Hystory of Science and Gynecology, CIBER en Epidemiología y Salud Pública (CIBERESP), Miguel Hernández University, Elche, Spain

**Keywords:** bladder, microbiota, cancer, actinobacteria, sequencing

## Abstract

Evidence suggests that microbiota may contribute to the pathogenesis of several diseases, including cancer. In the case of bladder cancer, preliminary studies have found alterations in the urinary microbiota of patients with urothelial carcinoma compared with healthy individuals. Conversely, the urinary microbiota differ between men and women, and it has been hypothesized that these differences are associated with the lower incidence of bladder cancers in women. The objective of this study was to characterize the bladder microbiota in paired samples of tumor and non-tumor mucosa of patients with malignant bladder neoplasia using next-generation sequencing. In addition, we aimed to study potential differences in microbial composition in tumor samples according to clinical and pathological variables, and to determine possible microbial profiles. We found significant differences in microbial richness at the genus level, with a higher richness observed in the non-tumor compared with the tumor mucosa. It was also shown that *Actinobacteria* were significantly more enriched in the non-tumor compared with the tumor mucosa (*P* = 0.014). In the multivariate analysis, we found significant differences in microbial composition according to tumor grade (*P* = 0.03 and 0.04 at the phylum and genus levels, respectively). Moreover, we detected a higher microbial richness in non-tumor vs. tumor tissues which agrees with the global assumption that microbial richness is an indicator of health. The greater abundance of members of the *Actinobacteria* phylum in the non-neoplastic bladder mucosa samples supports the hypothesis that a higher abundance of *Actinomycetes* is associated with a lower rate of bladder cancer in women and suggests a protective role for these microbiota. We detected a microbial profile that was enriched for *Enterococcus* in low-grade tumors. Finally, we identified the presence of two clusters in the microbial composition of the tumor mucosa samples, significantly enriched for the genera *Barnesiella*, *Parabacteroides*, *Prevotella*, *Alistipes*, and *Lachnospiracea_incertae_sedis* (Cluster 1), or *Staphylococcus* (Cluster 2). Further longitudinal studies are needed to assess the role of the bladder microbiota in carcinogenesis.

## Introduction

According to the latest GLOBOCAN (Sung et al., 2021) data, bladder cancer is the tenth most common cancer worldwide, with 573,278 new cases and 212,536 deaths annually. Moreover, the highest incidence rates of bladder cancer have been recorded in Europe. Globally, the incidence rates of bladder cancer are higher in men than in women (9.5 vs. 2.4) (Sung et al., 2021). In Spain, an estimated 12,200 new cases are diagnosed yearly, with 47 cases per 100,000 men and eight cases per 100,000 women (Burger et al., 2013). The etiological studies of bladder cancer have centered on several factors (American Society of Clinical Oncology, 2017), including smoking, which is the most important risk factor, host genetics, exposure to occupational chemicals, contaminated drinking water, infectious schistosomiasis, and other infectious diseases, such as urinary tract infections and viral infections (Gutiérrez et al., 2006; Burger et al., 2013; Cumberbatch et al., 2015; American Society of Clinical Oncology, 2017). Historically, this lower incidence of bladder cancer in women has been associated with a lower prevalence of smoking and lower occupational exposure to carcinogens. Smokers are considered to have a 2.5 times greater risk of developing this type of tumor than non-smokers (Babjuk, 2017). However, the increase in smoking among women has not led to an increase in the incidence of this malignant disease.

In the past, urine was considered sterile. Recently, the application of massive sequencing techniques revealed that the urinary microbiota differ between men and women (Fajkovic et al., 2011), and that these differences could be responsible for the lower incidence of bladder cancer in women. It was shown that microbiota rich in Actinomycetes could have a beneficial effect in preventing bladder cancer (Raoult, 2017). However, although these studies have analyzed urinary samples from patients with bladder cancer, there is little evidence related to the characterization of the bladder microbiota in patients with this malignancy. In addition, no study has focused on the clinical variables associated with the bladder microbiota, and this information could clarify how the microbiota affect human cancer pathogenesis, as well as its potential role as a prognosis marker. The goals of this study were to (i) describe the microbial taxonomic profiles of bladder tissues from patients with bladder cancer via a comparison between tumor and non-tumor tissue pairs and (ii) to elucidate the associations between bladder microbiota of tumor bearers and clinical and pathological variables.

## Materials and Methods

### Patient Cohort and Samples

Bladder cancer tumor samples were collected from 32 patients during surgical resection of radical cystectomy in the period from 2010 to 2017. Samples were collected after written, informed consent was obtained from the patients for the inclusion of surplus biological material in the Biobank of the General University Hospital of Elche (HGUE). Samples were processed and stored following standardized operation procedures. In brief, tumor samples were obtained from the central area of the neoplasia, avoiding selecting necrotic material and samples of bladder healthy mucosa were resected far from the neoplasia and distant from the surgical resection margins. Samples were immediately snap-frozen in isopentane and kept at −80°C until analysis. Samples were frozen within 30 min from surgery. This collection was requested for our study after complying with approval from the HGUE Ethics Committee (ethics approval date: June 2017). A total of 58 samples were included in the study, corresponding to 26 patients with paired samples (matched tumor and non-tumor samples) and six patients with only tumor tissue samples.

### Nucleic Acid Extraction

A pretreatment was carried out to eliminate the Optimal Cutting Temperature medium that was used to embed the samples. This process consisted of adding 1 ml of phosphate-buffered saline to the samples on dry ice, followed by centrifugation for 15 min at 1,000 *g* force removal of the supernatant, and addition of 180 μl of ATL Buffer (Tissue Lysis Buffer). After homogenization of the mixture, 20 μl of proteinase K was added. Finally, the samples were incubated at 56°C for 24 h with shaking, for their full digestion. For DNA extraction, DNeasy Blood and Tissue Mini Kits (Qiagen) were used according to the manufacturer’s instructions. Purified nucleic acids were quantified using a QuantiFluor^®^ dsDNA System fluorometer and stored at –80°C.

### Preparation of Samples and DNA Sequencing

Amplification of the 16S rDNA gene, including the V3–V4 region, was carried out according to the recommendations of the “16S Metagenomic Sequencing Library Preparation: Preparing 16S Ribosomal RNA Gene Amplicons for the Illumina MiSeq System” protocol. Illumina adapter overhang nucleotide sequences were added to the gene−specific sequences. The full-length primer sequences, using standard IUPAC nucleotide nomenclature, were: 16S Amplicon PCR Forward Primer, 5′-TCGTCGGCAGCGTCAGATGTGTATAAGAGACAGCCTAC GGGNGGCWGCAG-3′; and 16S Amplicon PCR Reverse Primer, 5′-GTCTCGTGGGCTCGGAGATGTGTATAAGAGAC AGGACTACHVGGGTATCTAATC-3′.

Polymerase chain reaction was performed as follows: 95°C for 3 min; followed by 25 cycles of 95°C for 30 s, 55°C for 30 s, and 72°C for 30 s; with a final cycle of 72°C for 5 min. The resulting libraries were indexed using the Illumina index kit [NextEra XT index kit (FC-121-1012)]. Libraries were normalized and grouped into a single sample. The size of the library was determined by automated electrophoresis using an Agilent Bioanalyzer 2100 system and an Agilent 5067-4626 High Sensitivity Kit.

Sequencing was performed on an Illumina MiSeq platform. Samples were loaded onto the MiSeq Reagent kit v3 300-cycle sequencing cartridge (MS-102-3003) and were sequenced using the option of paired readings of 2 × 300 bp according to the recommendations of the following protocols: “Preparing Libraries for Sequencing on the MiSeq^®^” and “Illumina’s MiSeq System Guide.” A negative control was used during sequencing, to ensure that no cross-contamination of prepared libraries occurred.

### Quality Control and Taxonomic Assignment

The raw sequenced reads were processed in the following way. First, quality was verified using the FastQC program (Andrews, 2010). Second, low-quality sequences were cleaned using the prinseq-lite program (Schmieder and Edwards, 2011) by applying the following parameters: min_length: 50, trim_qual_right: 30, trim_qual_type: mean, and trim_qual_window: 20. Forward and reverse reads that passed the quality check were joined using the FLASH program using default parameters (Magoč and Salzberg, 2011). Next, the Usearch (Edgar, 2016) program was used to remove chimeric sequences from the filtered reads. Finally, the Ribosomal Database Project database (Cole et al., 2014) was used to provide taxonomic assignments from phylum to genus. Taxonomic assignments with a score > 0.8 were selected.

### Statistical Analysis

Statistical analysis was carried out using the R programming language through Rstudio version 3.5.2 (R Core Team, 2018). We used several alpha (Shannon, Simpson, and Inverse Simpson diversity indeces and OBS, Chao2, and seChao2 richness indeces) and beta diversity metrics (Jaccard distance, Bray–Curtis distance, and generated principal coordinates analysis PcoA). The estimators of diversity and relative abundance were compared between the study groups using the Wilcoxon test for paired non-parametric data. The number of phyla and genera, as well as their proportion in the samples, were compared using the Wilcoxon test and the Mann–Whitney *U* test in the analyses of paired and unpaired samples, respectively. A two-dimensional multivariate analysis of principal components between groups and a permutational multivariate analysis of variance (PERMANOVA) test were performed to assess the association between clinicopathological variables and the composition of the microbiota. Intergroup differences at any taxonomic level were analyzed using the linear discriminant analysis (LDA), effect-size (LDA effect-size, LEfSe) method with the default settings (Segata et al., 2011). LefSe used the two-tailed non-parametric Kruskal–Wallis test to assess the significance of differences in OTUs between the two groups. An LDA was performed to estimate the effect size of the differentially abundant OTUs. The significance of differences was set at *P* < 0.05 and an LDA score (log_10_) > 2. A heatmap was prepared to identify the different clusters in the tumor samples. The clusters obtained were correlated with the tumor variables using the chi-squared test or Fisher’s test. A survival analysis was performed using the Kaplan–Meier estimator, to compare the various clusters identified.

## Results

### Characteristics of the Patients Included in the Study

Patient ages at the time of surgery ranged from 48 to 85 years (mean age, 67 years). Twenty-seven of the patients were male. Histologically, 22 tumors (68.75%) were urothelial and 29 (90.62%) were high grade. Postoperative staging showed that six tumors were stage 2; 13 and 13 tumors were stages 3 and 4, respectively; whereas no tumor was stage 1. The clinical variables included in the study were functional status according to the Eastern Cooperative Oncology Group scale and presence of any of the following comorbidities: diabetes mellitus, hypertension (HT), chronic obstructive pulmonary disease (COPD), and dyslipidemia and/or cardiovascular disease. Patient characteristics are given in greater detail in Table 1.

**TABLE 1:** Clinicopathological features of the patients included in this study.

	Patients (*n* = 32)
**DEMOGRAPHIC FEATURES**	
**Gender**	
Male	27
Female	5
**Age in years, mean (rank)**	67 (48–85)
**CLINICAL FEATURES**	
**DM, N (%)**	
No	26 (81.25)
Yes	6 (18.75)
**Hypertension, N (%)**	
No	20 (62.50)
Yes	12 (37.50)
**COPD, N (%)**	
No	24 (75.00)
Yes	8 (25.00)
**Dyslipidemia, N (%)**	
No	20 (62.50)
Yes	12 (37.50)
**Cardiovascular disease, N (%)**	
No	25 (78.12)
Yes	7 (21.88)
**Functional status according to ECOG scale, N (%)**	
0	2 (6.25)
1	12 (37.5)
2	5 (15.63)
3	1 (3.13)
Unknown	12 (37.5)
**TUMORAL FEATURES**	
**Histology, N (%)**	
Urothelial	22 (68.75)
Non-Urothelial	9 (28.13)
Unknown	1 (3.13)
**Grade, N (%)**	
Low	3 (9.38)
High	29 (90.62)
**Stage of tumor, N (%)**	
I	0 (0.00)
II	6 (18.75)
III	13 (40.63)
IV	13 (40.63)
**pT, N (%)**	
1	1 (3.12)
2	9 (28.13)
3	13 (40.62)
4	9 (28.13)
**Lymph node affectation (%)**	
No	17 (53.13)
Yes	11 (34.37)
Unknown	4 (12.5)
**Metastasis, N (%)**	
No	27 (84.38)
Yes	4 (12.50)
Unknown	1 (3.13)
**Lymphovascular invasion, N (%)**	
No	7 (21.88)
Yes	24 (75.00)
Unknown	1 (3.13)
**Death, N (%)**	
No	18 (56.25)
Yes	13 (40.62)
Unknown	1 (3.13)

### Sequencing Data

Samples were sequenced in duplicate to increase the precision of the study. The average number of high-quality sequences per sample was 27,332 (range: 3,084--149,206) and 13,378 (3,615--41,346), corresponding to replicates 1 and 2, respectively. An average of 20,335 sequences was obtained. The number of OTUs identified with an average relative abundance greater than 1% was: four phyla, 22 families, and 24 genera. The data for this study have been deposited in the European Nucleotide Archive (ENA) at EMBL-EBI under accession number PRJEB45435^1^.

### Differential Microbial Composition Between Matched Tumor and Non-tumor Samples

In this analysis, we only included 26 patients with paired samples (tumor mucosa vs. non-tumor mucosa), to compare bacterial diversity and to identify bacterial taxa with a significantly different abundance between the groups of samples. Both α-diversity and relative abundances of the taxonomic groups were calculated on various taxonomic ranks (from phyla to genera). Overall, the α-diversity indices were higher in the non-tumor mucosa than in the normal mucosa, with statistical significance detected at the phylum and genus level (Table 2). The most frequent phyla were *Firmicutes* (40.16% in tumor mucosa and 39.56% in non-tumor mucosa) and *Bacteroidetes* (29.15% in tumor mucosa and 28.17% in non-tumor mucosa), followed by *Proteobacteria* (22.96% in tumor mucosa and 21.35% in non-tumor mucosa) and *Actinobacteria* (6.18% in tumor mucosa and 9.05% in non-tumor mucosa), representing 98.45 and 98.13% of the microbiota of the tumor mucosa and non-tumor mucosa, respectively. Samples differed statistically regarding the relative abundance of *Actinobacteria* phylum (Table 3). Normal mucosa was enriched for *Actinobacteria* (9.05 vs. 6.18, *P* = 0.014).

**TABLE 2:** Comparison of α-diversity indices between tumor and matched normal tissues.

	Non-tumor	Tumor	*P-value
**Phylum**			
Simpson	0.66	0.61	**0.005**
Inverse Simpson	3.15	2.90	**0.005**
Shannon	1.27	1.17	**0.011**
OBS	7.50	6.88	0.135
Chao2	8.56	7.61	0.173
seChao2	1.67	1.30	0.324
**Family**			
Simpson	0.89	0.83	0.340
Inverse Simpson	12.01	11.58	0.548
Shannon	2.79	2.61	0.247
OBS	38.15	34.27	0.083
Chao2	46.25	42.24	0.105
seChao2	6.68	6.59	0.641
**Genus**			
Simpson	0.89	0.85	0.269
Inverse Simpson	12.55	12.46	0.617
Shannon	2.99	2.86	0.173
OBS	46.62	40.12	**0.012**
Chao2	56.45	49.84	**0.049**
seChao2	7.19	7.18	0.980

*P-values are from Wilcoxon test for paired samples. Bold values indicate statistical significance at the p < 0.05 level.

**TABLE 3:** Differences in the relative abundances of taxa stratified by type of tissue.

	Relative abundance (%)		*P*-value
	Non-tumor	Tumor	
**Phylum**			
Actinobacteria	9.05	6.18	**0.014**
Bacteroidetes	28.17	29.15	0.708
Firmicutes	39.56	40.16	0.353
Proteobacteria	21.35	22.96	0.745
**Genus**			
*Corynebacterium*	1.37	2.02	0.483
*Delftia*	1.82	1.41	0.303
*Enterococcus*	1.59	5.18	0.434
*Escherichia.Shigella*	6.28	6.63	0.565
*Faecalibacterium*	1.29	1.40	0.453
*Peptoniphilus*	1.53	0.85	0.515
*Propionibacterium*	4.49	2.91	0.084
*Staphylococcus*	4.42	6.06	0.165
Streptococcus	2.27	1.65	0.189
*Bacteroides*	16.27	16.66	0.708
*Aerococcus*	2.49	0.19	0.656
*Bifidobacterium*	1.68	0.74	0.534
*Lachnospiracea_incertae_sedis*	1.37	1.11	0.548
*Alistipes*	3.25	4.45	0.129
*Barnesiella*	1.23	1.51	0.199
*Clostridium.XlVa*	0.79	1.02	0.295
*Halanaerobacter*	1.09	0.79	0.923
*Parasutterella*	2.13	2.34	0.617
*Prevotella*	4.32	4.28	0.980
*Roseburia*	1.39	1.59	0.783
*Lactobacillus*	3.66	3.05	0.423
*Parabacteroides*	3.81	4.13	0.708
*Phascolarctobacterium*	5.19	4.42	0.515
*Desulfohalobium*	1.49	0.99	0.423

*Bold values indicate statistical significance at the p < 0.05 level.*

**FIGURE 1:**
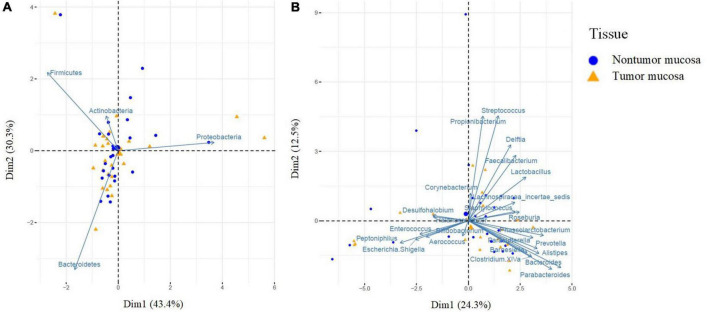
Principal component analysis (PCA) of bacteria at the phylum **(A)** and genus **(B)** level according to the type of tissue. PCA was performed using the Euclidean distance as a metric element and the Kaiser criterion to select the number of components. PCA shows the microbial community composition in relation to non-tumor mucosa (blue cicles, *n* = 26) and tumor mucosa (yellow triangles, *n* = 26) from bladder cancer patients with paired samples. Principal component (PC1 and PC2) together, account for 73.7% **(A)** and 36.8% **(B)** of the variance in these data.

We also analyzed the β-diversity. First, samples were compared to visualize the differences through non-metric multidimensional scales using the Jaccard and Bray–Curtis indices, for frequency and abundance, respectively. At the phylum and genus level, no differences were observed between the two groups (Supplementary Figure 1). A principal component analysis was performed using the Euclidean distance as a metric element and the Kaiser criterion to select the number of components. PC1 and PC2 accounted for 73.7% of the data variation (phylum component analysis). Figure 1A shows how the first main component was positively associated with *Proteobacteria* and negatively associated mainly with *Firmicutes* and *Bacteroidetes*, whereas the second main component was positively associated with Firmicutes and negatively associated with *Bacteroidetes*. A multivariate analysis was performed using the PERMANOVA test to assess whether the observed differences were significant, and no differences were found between the two groups (Supplementary Table 1). Finally, in the genus component analysis, PC1 and PC2 accounted for 36.8% of the data variation. Figure 1B shows how the first main component was positively associated with *Bacteroides* and *Parabacteroides* and negatively associated with *Peptoniphilus*, whereas the second main component was positively associated with *Propionibacterium*, *Streptococcus*, *Delftia*, and *Faecalibacterium*. In the multivariate analysis using the PERMANOVA test, no significant differences were observed between the tumor and non-tumor mucosa regarding microbial composition (Supplementary Table 1).

### Analysis of the Tumor Microbiota Based on Clinicopathological Variables

**FIGURE 2:**
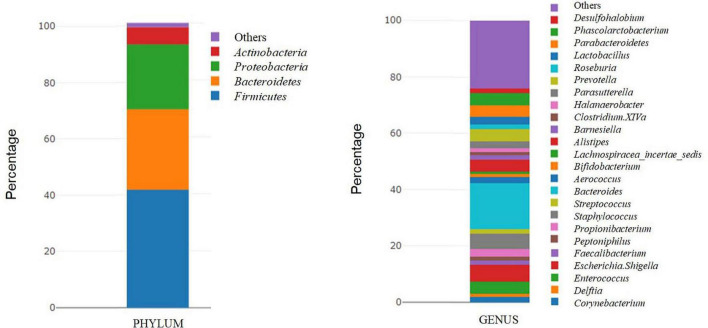
Taxonomic microbial composition detected in tumor samples. Relative abundance of bacterial communities in tumor tissues (*n* = 32) of bladdder cancer patientes are displayed at phylum level and genus level. Phyla and genera totaling <1% of samples were assigned as “Others.”

The samples used for this analysis were 32 tumor tissues from patients with bladder cancer. The most frequent phyla were *Firmicutes* (41.46%) and *Bacteroidetes* (28.23%), followed by *Proteobacteria* (22.78%) and *Actinobacteria* (6.06%), representing 98.53% of the total microbiota. At the genus level, the most frequent genera were *Bacteroides* (16.24%), *Escherichia-Shigella* (6.07%), *Staphylococcus* (5.43%), and *Enterococcus* (4.25%), representing 31.99% of the total microbiota (Figure 2).

**FIGURE 3:**
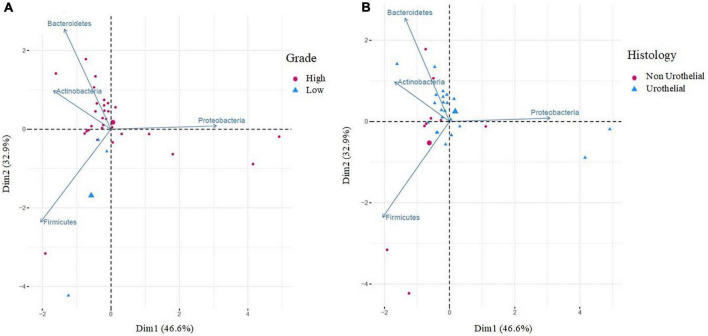
Principal component analysis (PCA) of bacteria according to the grade of the tumor **(A)** and histology **(B)**, at the phylum level. PCA **(A)** shows the microbial community composition of tumor tissues in relation to high grade (red cicles, *n* = 29) and low grade (blue triangles, *n* = 3). PCA **(B)** shows the microbial community composition of tumor tissues in relation to histology: non-urothelial (red cicles, *n* = 9) and urothelial (blue triangles, *n* = 22). Principal component (PC1 and PC2) together, account for 79.5% of the variance in these data.

**FIGURE 4:**
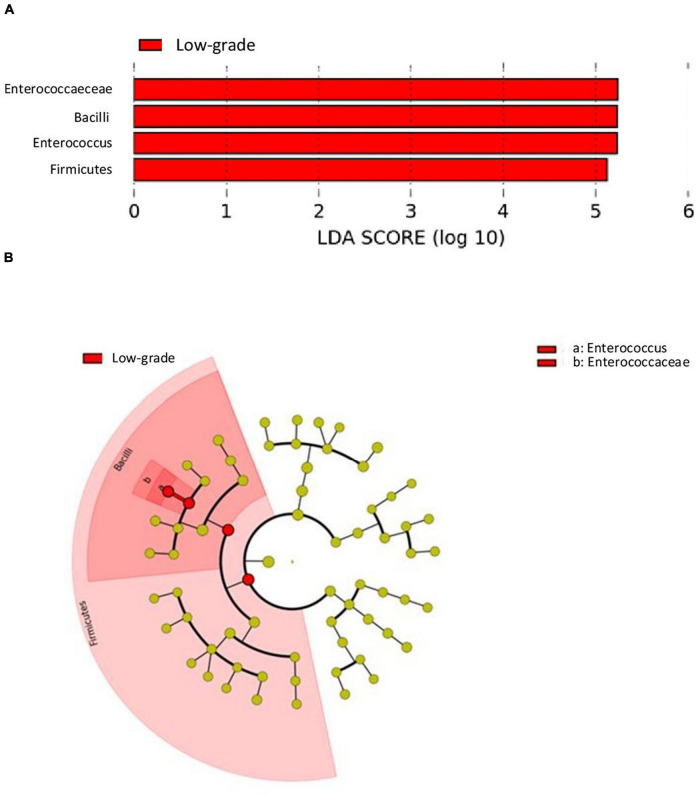
LDA effect size (LefSe) analysis showing a greater enrichment for Enterococcus in low-grade tumors. **(A)** The histogram of the Linear discriminant analysis (LDA) scores illustrating the differentially abundant taxa in the low-grade tumors. Horizontal bars represent the effect size for each taxon: red color indicates taxa enriched in low-grade tumors group and green color would indicate taxa enriched in high-grade tumors group (absent because there were no differentially abundant taxa in high-grade tumors). **(B)** The cladogram illustrating the different taxonomic level from inside to outside that are significantly more abundant in low-grade tumors group: the genus *Enterococcus* (a) and the family *Enterococcaceae* (b).

**FIGURE 5:**
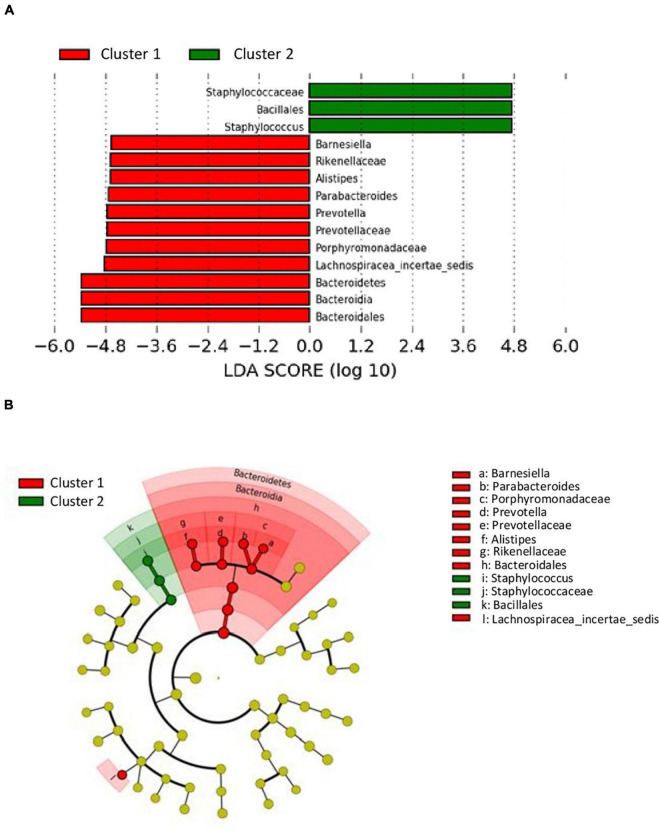
LDA effect size (LefSe) analysis showing differences of taxa between clusters. **(A)** The histogram of the Linear discriminant analysis (LDA) scores illustrating the differentially abundant taxa between clusters. Horizontal bars represent the effect size for each taxon: red color indicates taxa enriched in cluster 1 group and green color indicates taxa enriched in cluster 2 group. **(B)** The cladogram illustrating the different taxonomic level from inside to outside that are significantly more abundant in cluster 1 group (red) and cluster 2 group (green).

The analysis of differences in taxonomic abundance between samples based on clinical and tumor variables was performed using the multivariate analysis PERMANOVA (Table 4). Significant differences in the microbiota were observed according to the grade of the tumor at the phylum and genus level (*P* = 0.03 and 0.04, respectively) and the histology at the genus level (borderline statistical significance, *P* = 0.06). These differences in the microbiota were represented using Principal component analyses (PCAs). In the PCA of the phyla, PC1 and PC2 represented 79.5% of the variation in the data. The first main component was positively associated with *Proteobacteria* and negatively associated with *Actinobacteria*, *Firmicutes*, and *Bacteroidetes*, whereas the second main component was positively associated with *Bacteroidetes* and negatively associated with *Firmicutes*. Figure 3 shows the PCAs of the phyla according to the grade of the tumor (A) and the histology (B). In the PCA of the genera, PC1 and PC2 accounted for 39.6% of the data variation. The first main component was mainly positively associated with *Bacteroides*, *Parabacteroides*, and *Prevotella*, whereas the second main component was positively associated with *Peptoniphilus*, *Aerococcus*, and *Bifidobacterium*, and negatively associated with *Halanaerobacter* and *Desulfohalobium* (Supplementary Figure 2 shows the PCA according to the grade of the tumor at the genus level). An LEfSe analysis was performed to compare the estimated phylotypes of the patients with low and high tumor grades. The results showed differences in the phylogenetic distribution of the microbiota of both groups. The LDA scores indicated that the relative abundances of *Enterococcus* were much more enriched in the low-grade patients than they were in the high-grade patients (Figure 4). A heatmap was prepared to compare the relative abundances of the genera of the tumor mucosa samples, to identify different clusters. Three clusters were observed. The results of the LEfSe analysis between the clusters are shown in Figure 5. The LDA scores indicated that the relative abundances of *Barnesiella*, *Parabacteroides*, *Prevotella*, *Alistipes*, and *Lachnospiracea_incertae_sedis* were much more enriched in Cluster 1 patients, whereas the relative abundances of *Staphylococcus* were more enriched in Cluster 2 patients. Once the clusters were identified, their possible correlation with the tumor variables was analyzed. The variables “histology” and “lymph node involvement” bordered on statistical significance (*P* = 0.09 and *P* = 0.06, respectively). Finally, a survival analysis was performed using the Kaplan–Meier estimator to compare the survival of the patients in months according to clusters. Supplementary Figure 3 shows how clustering of patients was not a significant prognostic factor for survival (*P* = 0.4).

**TABLE 4:** Results of the permutational multivariate analysis of variance (PERMANOVA) of the clinicopathological variables.

	PERMANOVA	
Variable	Phylum *P*-value	Genus *P*-value
Gender	0.53	0.39
DM	0.20	0.50
Hypertension	0.55	0.38
COPD	0.90	0.77
Dyslipidemia	0.71	0.95
Cardiovascular disease	0.96	0.68
Histology	0.06	0.08
**Grade**	**0.03**	**0.04**
Stage of tumor	0.09	0.68
pT	0.52	0.24
Lymph node affectation	0.58	0.34
Metastasis	0.12	0.13
Lymphovascular invasion	0.85	0.98

*Bold values indicate statistical significance at the p < 0.05 level.*

## Discussion

We observed a higher α-diversity in the non-tumor mucosa samples than in the tumor mucosa samples of patients with bladder cancer. Statistically significant differences were observed for the Shannon (*P* = 0.011) and Simpson (*P* = 0.005) diversity indices and the richness indices [OBS (*P* = 0.012) and Chao (*P* = 0.049)]. This greater microbial biodiversity of non-tumor tissues than tumor tissues fits well with the global assumption that species richness is an indicator of healthy microbiota. However, there is great controversy in the comparative analysis of the microbiota according to the types of samples and/or the health state of the patients. Our results coincided with other studies carried out in patients with colorectal cancer (CRC), which revealed that the tumor samples had a lower biodiversity than the colorectal mucosa of healthy volunteers (Geng et al., 2013; Gao et al., 2015). In contrast, other studies showed a similar microbial richness and diversity between the tumor and non-neoplastic mucosa (Leung et al., 2019). This lack of consistency in the published results could be explained by the heterogeneity in the design of the various studies, the variations between the groups of subjects, and, in the majority of the cases, the low statistical power resulting from the small sample sizes of the studies.

Popović et al. (2018) evaluated α-diversity in patients with urothelial cancer and found no statistically significant differences between urine samples from patients with cancer and those from healthy volunteers (species richness and Simpson index) (Popović et al., 2018). In contrast, Wu et al. (2018) observed a significant increase in bacterial richness [observed species index (*P* = 0.008), Chao index (*P* = 0.008), and Ace index (*P* = 0.003)] in urinary samples from patients with bladder cancer (Wu et al., 2018), and an increase in microbial richness in the high-risk group of recurrence and progression compared with the low-risk group. These differences could be explained by the fact that those authors made comparisons in urine samples. Urine samples are usually used in studies of urothelial cancer because of their non-invasive nature. To our knowledge, only one recently published study (Liu et al., 2019) analyzed the microbiota in bladder tissues. In that study, the researchers analyzed 12 tumor tissue biopsy samples and their corresponding samples of adjacent non-tumor tissue. They observed statistically significant differences in the Shannon diversity index between cancerous and non-cancerous tissues (*P* = 0.0417), with significantly less diversity being observed in tumor tissues than non-tumor tissues. However, they did not observe statistically significant differences between the richness indices (Ace and Chao) and detected a lesser degree of species richness and diversity in tumor tissues, as shown here.

Accumulated evidence supports the role of the microorganisms that inhabit the urinary tract in the pathophysiology of urological diseases. Urinary microbiota are different between the healthy population and patients with urological disorders. Clinical and epidemiological studies have suggested an association between infectious agents and the process of carcinogenesis (Kostic et al., 2012; Lu et al., 2014; Garrett, 2015; Neto et al., 2015; Alfano et al., 2016). Conversely, host bacteria of the urinary microbiome can control the pathogenic growth of potentially harmful bacteria in the genitourinary tract, similar to the prevention of vaginal infections afforded by *Lactobacillus*. Despite this evidence, it is not well defined whether the urinary microbiome influences the development or progression of bladder cancer. Most of the studies on this topic have focused on urinary microbiota, whereas the bladder microbiota have not been explored. In our study, we analyzed the microbiota of the bladder in tissue samples from cystectomized patients after a diagnosis of bladder carcinoma; therefore, we could not analyze the bladder microbiome in a healthy population. There are no available data in the literature for comparison with our results because the bladder ecosystem itself has not been explored. Studies in healthy populations (Thomas-White et al., 2018) or those that analyzed the possible association between the microbiota and urological disorders (Pearce et al., 2014; Curtiss et al., 2017) evaluated the bladder microbiota in urine samples.

We found that the most abundant phyla were *Firmicutes* and *Bacteroidetes*, followed by *Proteobacteria* and *Actinobacteria*. According to previous evidence, the urinary microbiome is also mainly represented by these phyla, both in healthy populations and in patients with bladder cancer. *Firmicutes* and *Actinobacteria* are the most abundant phyla in healthy populations (Pearce et al., 2014; Popović et al., 2018; Thomas-White et al., 2018; Bi et al., 2019) and in patients with bladder cancer (Popović et al., 2018; Bi et al., 2019), although other authors reported a greater dominance of the phylum *Proteobacteria* in both groups of subjects (Wu et al., 2018).

At the genus level, there are also discrepancies between the different studies (Xu et al., 2014; Popović et al., 2018; Bi et al., 2019). The high degree of interindividual variability could be a possible explanation for these differences. It is important to highlight that many of these studies were carried out in samples of populations with marked cultural and nutritional differences, and that epidemiological factors, such as diet, together with genetic factors, have a great effect on microbiota. Therefore, additional studies are needed to assess the agreement between the two ecosystems.

In our study, we found a significantly higher abundance of *Actinobacteria* (*P* = 0.014) in the samples of non-neoplastic bladder mucosa than in tumor tissues. This result strengthens the hypothesis initially proposed that microbiota rich in *Actinomycetes* could be related to the lower incidence of bladder cancer in women (Raoult, 2017) and, therefore, could have a preventive effect, similar to the BCG vaccine (composed of Actinomycetes), which has a protective effect in the treatment and prevention of relapses of bladder cancer (Whiteside et al., 2015).

Our results showed for the first time an increase in *Actinobacteria* in non-tumor mucosa samples compared with tumor samples. Although statistical significance was not reached at the genus level, *Propionibacterium* exhibited a higher relative abundance in the non-tumor mucosa (*P* = 0.08). *Propionibacterium* and *Bifidobacterium* (also with greater abundance in non-neoplastic mucosa samples in our study, albeit without significant differences) are bacteria that are closely related to healthy microbiota. Specifically, *Propionibacterium freudenreichii* is a commercially relevant bacterium because of its potential as a probiotic, its use as a protective agent against CRC has been described. According to the results obtained by Casanova et al. (2018), this bacterium could be used as a probiotic in the prevention of CRC in the early stages of the carcinogenesis process (Casanova et al., 2018).

Liu et al. (2019) analyzed 12 cases of paired samples and found that *Actinobacteria* were overrepresented in cancerous tissues (Liu et al., 2019). Other than this study, no analyses of microbiota in tissue samples have been reported. However, other authors have observed differences in the urinary microbiota between healthy patients and patients with bladder cancer (Xu et al., 2014; Popović et al., 2018; Wu et al., 2018; Markowski et al., 2019). To explain these differences, in addition to the high degree of interindividual variability mentioned previously, we must highlight the small sample size of some of these works (Xu et al., 2014; Popović et al., 2018; Liu et al., 2019).

We carried out a multivariate analysis to assess if there were differences in the microbial composition according to the type of tissue; no significant differences were found at any of the taxonomic levels. Thus, we concluded that the two types of samples had a similar microbial profile. These results were consistent considering that these were paired samples from the same patients. However, the PCA revealed a microbial profile consisting of *Propionibacterium*, *Streptococcus*, *Delftia*, and *Faecalibacterium* in certain samples. Considering the presence of *Faecalibacterium* in the described microbial profile, which is considered as a health biomarker, it is possible that the possible protective effect attributed to *Propionibacterium* in the univariate analysis did not stem from a specific microorganism (*Propionibacterium*); rather, it may have been the result of a microbial community, as occurs with the relationship between the microbiota and CRC. Previous evidence supports the association between the neoplastic process and specific microorganisms, such as *Fusobacterium nucleatum* (Kostic et al., 2013) or *Streptococcus gallolyticus* (Boleij et al., 2011) (pro-oncogenic microbiota); therefore, it seems that the effect is also associated with a procarcinogenic dysbiotic intestinal microbial community (Tjalsma et al., 2012). Furthermore, it has been postulated that alterations in colorectal metabolism resulting from bacterial dysbiosis could lead to insufficient production of metabolites involved in pathways that contribute to protection against CRC progression (Tjalsma et al., 2012). *F. prausnitzii* is one of the major components of the gut microbiota and the most important butyrate-producing bacterium in the colon. The decrease in this commensal bacterium favors inflammatory processes and has been negatively correlated with CRC (anti-oncogenic microbiota) (Ferreira-Halder et al., 2017).

Our second aim involved identifying differences in the tumor microbiota in association with clinicopathological variables. First, we carried out a multivariate analysis of the microbial composition according to sex. Studies carried out using molecular biology techniques have shown that the microbiota of the genitourinary system is different between women and men (Qin et al., 2010; Fouts et al., 2012). In both cases, *Firmicutes* are predominant; however, the urine of women shows a greater diversity and a greater abundance of *Actinobacteria* and *Bacteroidetes* (Lewis et al., 2013). At the genus level, the healthy urinary microbiome is characterized by a predominance of *Lactobacillus* in women and *Corynebacterium* in men (Fouts et al., 2012). As commented above, these differences suggest that the composition, diversity, or abundance of certain microorganisms in the female genitourinary ecosystem provides a level of protection against the development of bladder cancer (Markowski et al., 2019). In general, studies that evaluated the microbiota of individuals with and without bladder cancer have included men exclusively (Popović et al., 2018; Wu et al., 2018; Liu et al., 2019). We included 58 samples from 32 patients, 27 men and five women (5.4 men for each woman), with a median age of 67 (48–85) years. Although we considered this to be a representative population in terms of sex and age (according to the Spanish Association Against Cancer, the male:female ratio is 3.8 in Europe and 7 in Spain, and the majority of cases diagnosed in Spain are between 65 and 75 years of age), the multivariate analysis did not reveal significant differences between the two groups of patients, maybe because of the small size of the group of women. In addition, we were able to verify that the significant differences in the relative abundance of the phylum *Actinobacteria* (more abundant in the non-tumor vs. the tumor mucosa, *P* = 0.014) remained significant (*P* = 0.012, data not shown in the section “Results”) when samples corresponding to women were removed from the analysis.

We also evaluated the possible association between the tumor tissue microbiota and the clinical and pathological variables. No significant differences were observed according to the different clinical subgroups, i.e., diabetes, HT, COPD, dyslipidemia, and/or cardiovascular disease, whereas there were statistically significant differences in the microbiota depending on the grade of the tumor [both at the phylum and genus level (*P* = 0.03 and 0.04, respectively)]. Recent evidence suggests that there is a bidirectional relationship between intestinal dysbiosis and local and systemic inflammation, as well as between proinflammatory processes and alterations in the microbiota. Therefore, the association with the grade of the tumor seems especially relevant in this context. In the work of Liu et al. (2019), which studied tumor biopsies (Liu et al., 2019), and in the study reported by Wu et al. (2018), which used urine samples from patients with cancer (Wu et al., 2018), no significant differences in microbial composition were observed according to the grade of the tumor. In contrast, the latter authors observed differences in the composition of the urinary microbiota according to the degree of recurrence and progression.

Our results showed that the low-grade tumor mucosa had microbiota enriched in the genus *Enterococcus*. This result should be confirmed at the species level, to assess its clinical significance and potential procarcinogenic role. Given the ability of *Enterococcus faecalis* bacteria to produce extracellular superoxide at high concentrations, thus causing damage to cellular DNA (Huycke et al., 2001; Huycke and Moore, 2002), a greater abundance of this microorganism in low-grade tumors suggests its implication in the initial development of the tumor. However, quantitative studies at the species level would be necessary to confirm this hypothesis.

Finally, our patients were grouped into three clusters based on the similarity of the microbiota in the tumor mucosa, differentiating three different microbial profiles. Cluster 1 patients (13 patients) exhibited microbiota rich in *Bacteroidetes*, with the *Barnesiella*, *Parabacteroides*, *Prevotella*, and *Alistipes* genera predominating; Cluster 2 patients (12 patients) had microbiota rich in *Firmicutes*, with a predominance of the *Staphylococcus* genus; and Cluster 3 patients (six patients) did not show significant differences, probably because of the small sample size. The comparison of the different clusters with the clinicopathological variables revealed an absence of significant differences, although it was observed that the patients in Cluster 2 had a greater survival than did those in Cluster 1 (without significant differences).

This study had several limitations. First, the cross-sectional design did not allow the establishment of a causal relationship between the microbiota and the clinical and pathological variables analyzed. In addition, there was no control group because of the difficulty in obtaining a biopsy of the bladder tissue in the healthy population. Furthermore, we could not collect other possible confounding variables, such as tobacco use, occupational exposure, and the consumption of antibiotics. Nevertheless, we consider that our results generate hypotheses that must subsequently be contrasted with other types of longitudinal studies.

## Conclusion

The greater α-diversity observed in the non-tumor mucosa of patients with bladder cancer compared with the paired tumor mucosa, agrees with the global assumption that richness is an indicator of the health of microbiota. Here, we reported for the first time a significantly higher abundance of *Actinobacteria* in non-neoplastic bladder mucosa samples than in tumor tissues. This supports the hypothesis that microbiota rich in *Actinomycetes* are related to the lower incidence of bladder cancer in women and, therefore, may have a preventive effect against this type of cancer. The microbial composition of the tumor tissue showed the presence of significant differences according to the tumor grade, as low-grade tumors exhibited a microbial profile that was characterized by a higher enrichment for *Enterococcus*. In our opinion, these data should be confirmed at the species level to evaluate their clinical significance and potential procarcinogenic role. Finally, the differences observed between the tumor mucosa with respect to its paired non-tumor mucosa and the clinicopathological variables must be confirmed by longitudinal prospective studies, which will allow the establishment of the causal effect of the microbiota in the carcinogenesis process.

## Data Availability Statement

The datasets presented in this study can be found in online repositories. The names of the repository/repositories and accession number(s) can be found below: www.ncbi.nlm.nih.gov/, PRJEB4543.

## Ethics Statement

The studies involving human participants were reviewed and approved by General University Hospital of Elche Ethics Committee. The patients/participants provided their written informed consent to participate in this study.

## Author Contributions

MP-G and VS-H designed the research. MP-G carried out the nucleic acid extraction and bioinformatics analysis and was a major contributor to manuscript preparation. BL involved in the study design. MO-A involved in the clinical aspects of the study. ML and AS involved in sample storage and DNA extraction. LM-P and GD’A carried out the sequencing of samples and provided support in the bioinformatics analysis. RR-M involved in the data analyses. VS-H involved in data analysis and was a major contributor to manuscript writing. All authors read and approved the final manuscript.

## Conflict of Interest

The authors declare that the research was conducted in the absence of any commercial or financial relationships that could be construed as a potential conflict of interest.

## Publisher’s Note

All claims expressed in this article are solely those of the authors and do not necessarily represent those of their affiliated organizations, or those of the publisher, the editors and the reviewers. Any product that may be evaluated in this article, or claim that may be made by its manufacturer, is not guaranteed or endorsed by the publisher.

## Footnotes

1
https://www.ebi.ac.uk/ena/browser/view/PRJEB45435

## Supplementary Material

Supplementary Figure 1 Jaccard and Bray–Curtis indices at the phylum **(A)** and genus **(B)** level. Non-metric multidimensional scaling (NMDS) plot based on Jaccard (frequency) and Bray–Curtis (abundance) indices show no differences between non-tumor mucosa (blue, *n* = 26) and tumor mucosa (yellow, *n* = 26) from bladder cancer patients with paired samples.

Supplementary Figure 2 Principal component analysis (PCA) of bacteria according to tumor grade at the genus level. PCA shows the microbial community composition of tumor tissues in relation to high grade (red cicles, *n* = 29) and low grade (blue triangles, *n* = 3). Principal component (PC1 and PC2) together, account for 39.6% of the variance in these data.

Supplementary Figure 3 Survival analysis estimates of the different clusters detected in tumor tissues. Kaplan-Meier survival analysis of bladder cancer patients according to Cluster 1 in red (significantly enriched for the genera *Barnesiella, Parabacteroides, Prevotella, Alistipes*, and *Lachnospiracea_incertae_sedis*), Cluster 2 in blue (significantly enriched for the genera *Staphylococcus*) and Cluster 3 in green (no significantly enriched) shows a better survival for patients in Cluster 2 with no statistically significant differences (*P* = 0.4).
