# Supplementary material for: Profiling the Bladder Microbiota in Patients With Bladder Cancer
Source: Front Microbiol. 2022 Feb 7;12:718776. doi: 10.3389/fmicb.2021.718776 (PMC8859159; doi:10.3389/fmicb.2021.718776)
Supplement: Supplementary file 1 [file Table_1.docx]

**Supplementary Table S1.** Permutational multivariate analysis of variance (PERMANOVA) of bladder microbiota from tumor and matched normal tissues at the phylum and genus level.

| **Phylum** | **Df** | **SumOfSqs** | **R^2^** | **F** | **Pr (>F)** |
| --- | --- | --- | --- | --- | --- |
| Tissue | 1 | 159 | 0.00476 | 0.2392 | **0.826** |
| Residual | 50 | 33259 | 0.99524 |  |  |
| Total | 51 | 33419 | 1.00000 |  |  |

| **Genus** | **Df** | **SumOfSqs** | **R^2^** | **F** | **Pr (>F)** |
| --- | --- | --- | --- | --- | --- |
| Tissue | 1 | 379 | 0.01065 | 0.5382 | **0.876** |
| Residual | 50 | 35195 | 0.98935 |  |  |
| Total | 51 | 35574 | 1.00000 |  |  |
