# Supplementary figures and images for: Profiling the Bladder Microbiota in Patients With Bladder Cancer
Source: Front Microbiol. 2022 Feb 7;12:718776. doi: 10.3389/fmicb.2021.718776 (PMC8859159; doi:10.3389/fmicb.2021.718776)

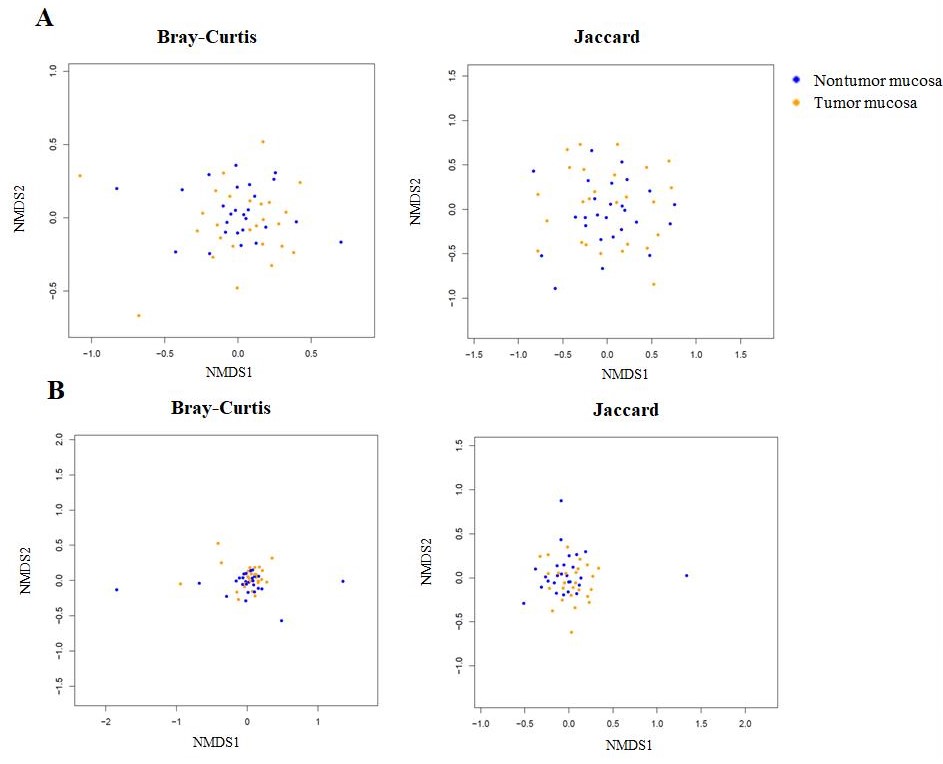

Supplement: Supplementary Figure 1 — Jaccard and Bray–Curtis indices at the phylum (A) and genus (B) level. Non-metric multidimensional scaling (NMDS) plot based on Jaccard (frequency) and Bray–Curtis (abundance) indices show no differences between non-tumor mucosa (blue, n = 26) and tumor mucosa (yellow, n = 26) from bladder cancer patients with paired samples. [file Image_1.JPEG]

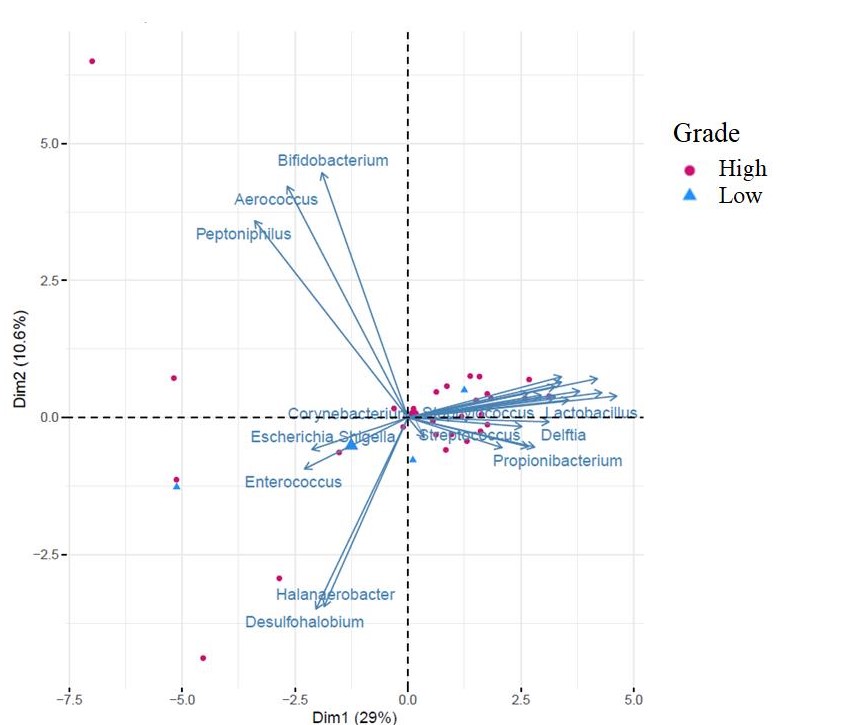

Supplement: Supplementary Figure 2 — Principal component analysis (PCA) of bacteria according to tumor grade at the genus level. PCA shows the microbial community composition of tumor tissues in relation to high grade (red cicles, n = 29) and low grade (blue triangles, n = 3). Principal component (PC1 and PC2) together, account for 39.6% of the variance in these data. [file Image_2.JPEG]

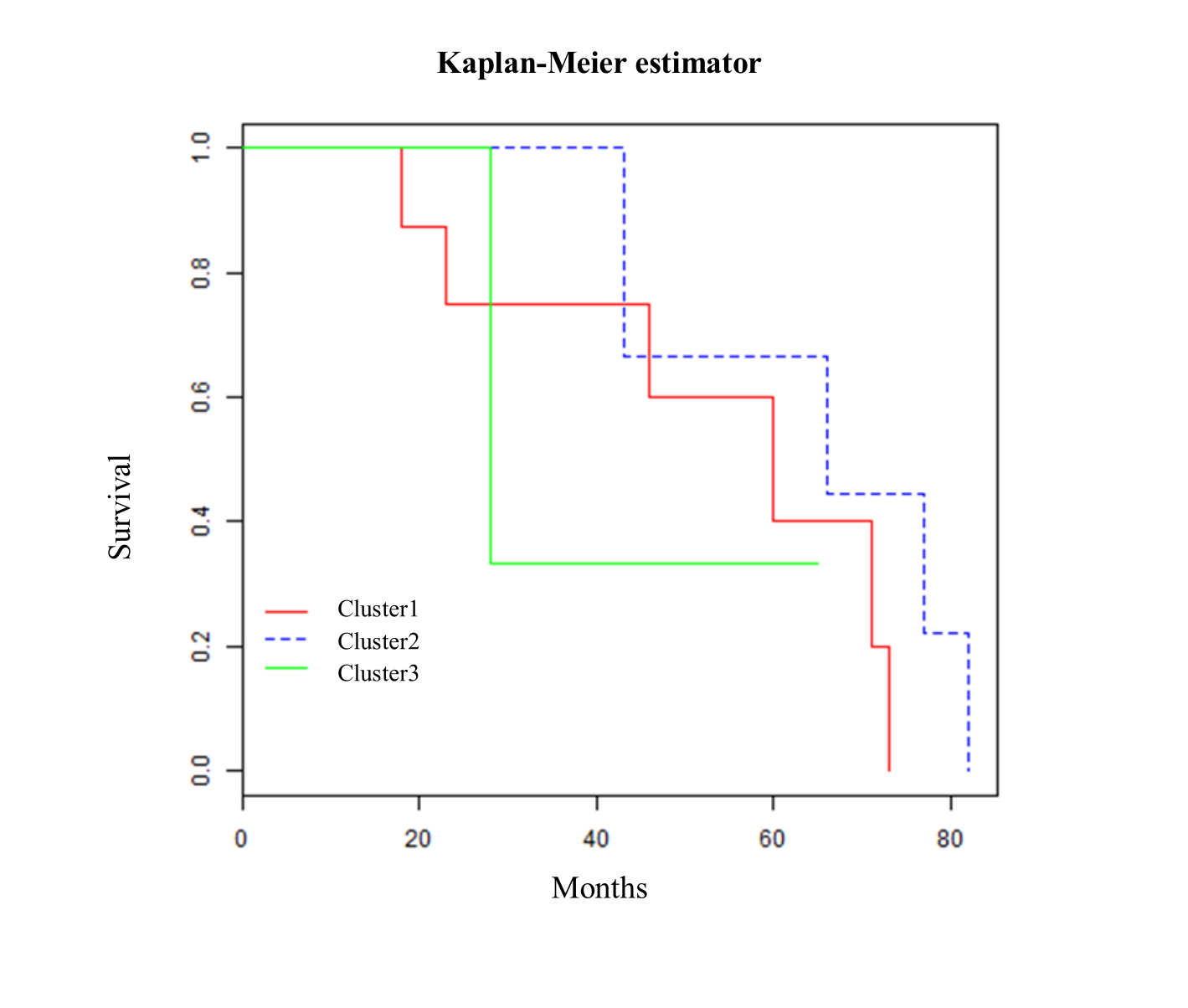

Supplement: Supplementary Figure 3 — Survival analysis estimates of the different clusters detected in tumor tissues. Kaplan-Meier survival analysis of bladder cancer patients according to Cluster 1 in red (significantly enriched for the genera Barnesiella, Parabacteroides, Prevotella, Alistipes, and Lachnospiracea_incertae_sedis), Cluster 2 in blue (significantly enriched for the genera Staphylococcus) and Cluster 3 in green (no significantly enriched) shows a better survival for patients in Cluster 2 with no statistically significant differences (P = 0.4). [file Image_3.JPEG]
